# Spindle Cell Carcinoma (Sarcomatoid) of Colon Presenting as Weight Loss

**DOI:** 10.7759/cureus.18146

**Published:** 2021-09-20

**Authors:** Mimoza Isufi, Jamila Begum Jabar Ali, Deepu Joseph, Tahani M Abugoukh, Naglaa G Ghobriel

**Affiliations:** 1 Pathology and Laboratory Medicine, University of Tirana, Faculty of Medicine, Albania, Waterbury, USA; 2 Internal Medicine, California Institute of Behavioral Neurosciences & Psychology, Fairfield, USA; 3 Internal Medicine, Davao Medical School Foundation, Davao, PHL; 4 Medicine, Shendi University, Cedar Rapids, USA; 5 Internal Medicine, University of Alexandria, Alexandria, EGY

**Keywords:** spindle cell carcinoma, sarcomatoid, colon, weight loss, adenocarcinoma

## Abstract

Sarcomatoid carcinoma of the colon is an extremely rare tumor. To date, less than 50 cases have been reported in the literature. It is rapidly growing, with a high recurrence rate, and has a very poor prognosis. Herein we present a 34-year-old male with a two-month history of weight loss, abdominal distention, and chronic history of constipation. Endoscopy was done and revealed undifferentiated adenocarcinoma of the transverse colon. On histopathology, the tumor was composed of sarcomatous and carcinomatous components. On immunohistochemistry, strong immunoreactivity for cytokeratin was found and the spindle cell component was largely vimentin positive.

## Introduction

Various terms have been used to describe sarcomatoid carcinomas, including pseudo sarcomatous carcinoma, carcinosarcoma, spindle cell carcinoma, and carcinoma with mesenchymal stroma [1].

With an uncertain histogenesis, sarcomatoid carcinoma is made up of mixed carcinoma cells and mesenchymal cells. This is an uncommon histopathological entity. Within the last century, sarcomatoid carcinomas have been discovered at various locations, such as the respiratory tract, female reproductive tract, and head and neck [2, 3]. The esophagus and oropharynx are the most frequently affected areas of the gastrointestinal tract [4]. The presence of this tumor in the colon is a rare occurrence. This may be because this tumor is markedly similar to malignant mesenchymal tumors, including gastrointestinal stromal tumor (GIST), malignant fibrous histiocytoma (MFH), and leiomyosarcoma. Diagnosis of sarcomatoid carcinoma requires the identification of squamous neoplastic components and epithelial differentiation of the spindle cell. Multiple cytokeratin and epithelial markers show positive staining in the spindle cells but up to 30% of spindle cell carcinoma (SpCC) will not stain for cytokeratin in the spindle cell component. Colon spindle cell carcinoma is diagnosed based on immunohistochemistry like cytokeratin and vimentin staining.

In this study, we present the case of a 34-year-old male patient with sarcomatoid carcinoma with presenting symptoms of weight loss and abdominal distention for the past two months.

## Case presentation

A 34-year-old male patient presented to our hospital with complaints of four episodes of nausea and vomiting for two days. He also reports abdominal distention and constipation for two months, fatigue, low-grade fever, and weight loss of 5 lb over these two months. He is non-hypertensive, non-diabetic. The patient has smoked a pack of cigarettes daily for the past 10 years. Family history and past medical history are unremarkable. 

**Figure 1:**
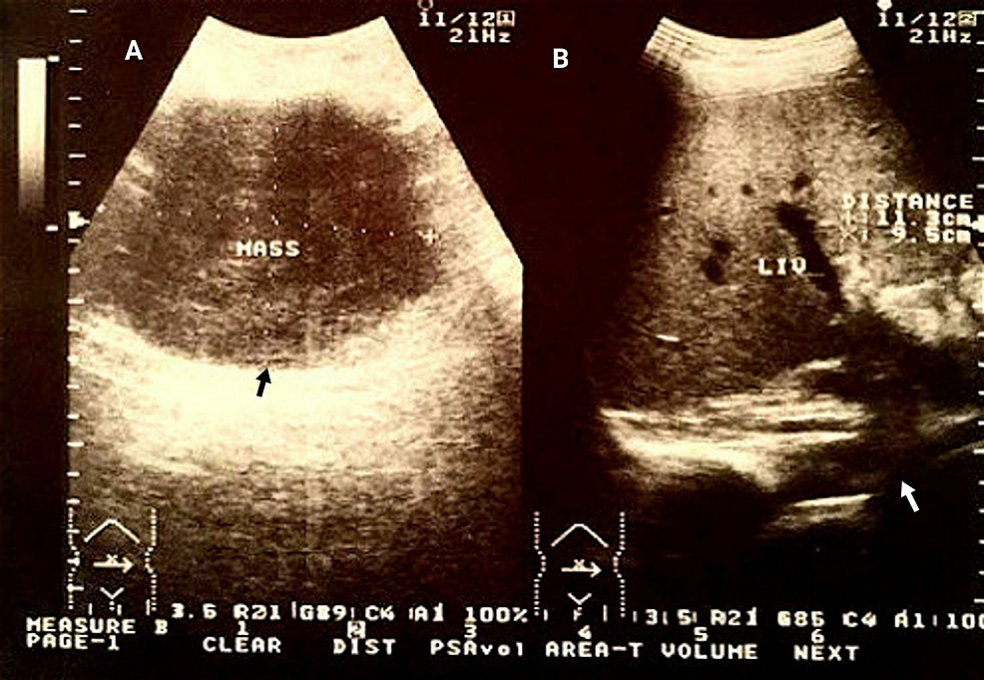
Ultrasound of the abdomen A: Black arrow showing a large mass in the abdomen;  B: White arrow showing accumulation of ascitic fluid in the abdominal cavity.

On physical examination the patient was pale and the abdomen was asymmetrically enlarged with a bulge in the epigastric and umbilical region, which was tender to deep palpation. The rest of the examination was normal. He was vitally stable with a blood pressure of 130/80 mmHg, pulse 83/min, oxygen saturation (SpO2) of 95% at room air, and random sugar of 134 mg/dl. Laboratory investigation showed low hemoglobin of 9 mg/dl, elevated serum levels of carcinoembryonic antigen and carbohydrate antigen 19-9. The abdominal sonogram showed a large mass in the pelvic cavity with ascites (Figure 1).

**Figure 2:**
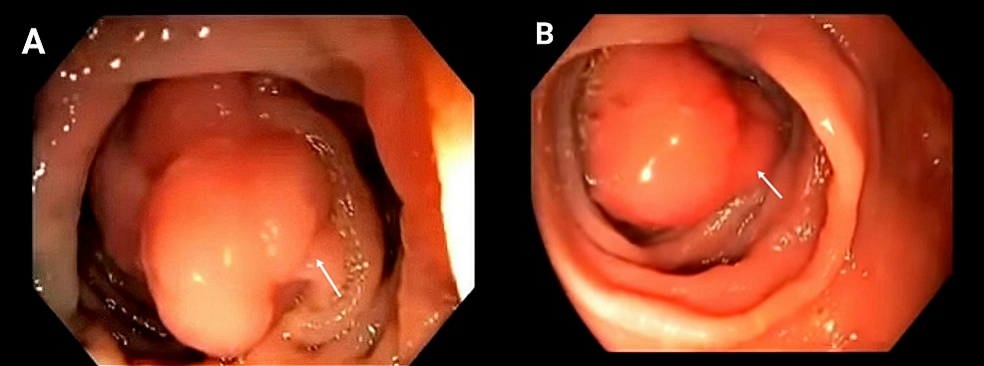
Endoscopic examination showing undifferentiated adenocarcinoma of the transverse colon A: White arrow showing the sessile lesion;  B: White arrow showing the polypoid lesion.

**Figure 3:**
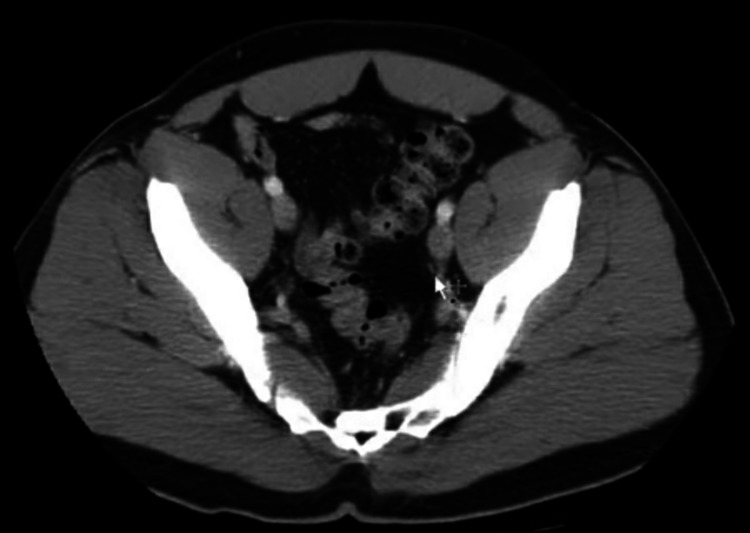
CT scan of the abdomen and pelvis 10mm axial section through the abdomen and pelvis with oral and non-ionic low osmolar IV contrast: white arrow showing no adjacent metastasis.

The endoscopic examination at admission revealed undifferentiated adenocarcinoma of the transverse colon (Figure 2). Abdominal ultrasound was done to locate the position of the tumor and adjacent structures. Moreover, a color doppler was done to rule out any aortic abnormality. On abdominal CT, no adjacent metastasis was found (Figure 3)

**Figure 4:**
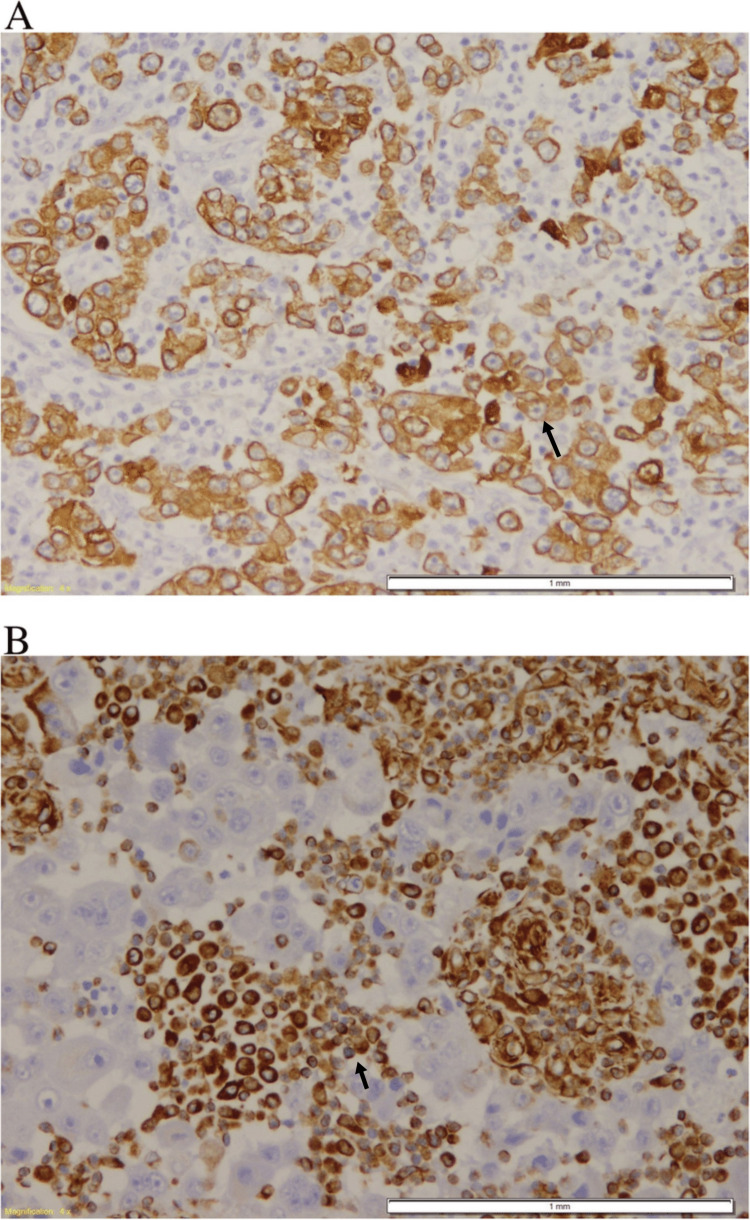
Immunohistochemical stain showing strong immunoreactivities for cytokeratin and vimentin A: Black arrow shows positive cytokeratin antibody in the epithelial cells;  B: Black arrow shows positive vimentin antibody in the mesenchymal component.

Resection of omentum measuring 21.0cm*18.0cm*11.5cm was done which showed a fungating mass in the transverse colon with tumor cells invading serosal tissue adjacent to the colon. No lymph node involvement was found. On histopathology, the tumor was composed of the sarcomatous and carcinomatous components. On immunohistochemistry, strong immunoreactivities for cytokeratin were found, and the spindle cell component was largely vimentin-positive (Figure 4a, 4b).

His tumor was classified as stage 2B according to TNM staging. The patient preferred to undergo surgical excision. Laparotomy was performed and all the dysplastic areas were removed. Moreover, he also received three cycles of 5-fluorouracil and leucovorin after surgery.

## Discussion

Sarcomatoid carcinoma is an aggressive and rare tumor commonly arising in the female genital tract and the head and neck [5]. The upper aerodigestive tract including the esophagus and stomach are sometimes involved. The prognosis is very poor and prognostic factors remain poorly understood. However, since the first report of colonic sarcomatoid carcinoma by Weidner and Zekan [6]. The clinical course of sarcomatoid carcinomas is aggressive and patients frequently present with signs and symptoms associated with distant metastasis. According to our knowledge, 23 cases of sarcomatoid carcinomas and colon carcinomas have been reported in the literature to date [7].

Both mesenchymal and epithelial components are present in histological samples of sarcomatoid carcinomas. High-grade adenocarcinoma is the main epithelial component of these tumors, whereas a spindled appearance with varying degrees of mesenchymal-like differentiation is described in the sarcomatous component. Despite the arrival of electron microscopy and immunohistochemistry, the nomenclature of these bi-differentiated tumors has remained a debatable topic. Historically, the condition in which the spindle cells were benign and the epithelial component was malignant has been described using the term pseudosarcoma [8].

The relation between tumor progression from the carcinomatous to the sarcomatous phase and the existence of productive retroviral infection in the sarcomatous cell was described by Gentile et al [9]. Delahunt et al. [10] reported that the phenotypic conversion of carcinoma into sarcomatoid tissue is linked with the progressive accumulation of p53 proteins, therefore demonstrating that dedifferentiated tumor cells with p53 mutations have increasing clonal dominance in them.

A specific treatment guideline does not exist as the number of cases of colonic sarcomatoid carcinoma is limited. However, an acceptable approach may be radical surgery adjunct with chemotherapy and a close follow-up [11].

## Conclusions

To summarize, sarcomatoid carcinoma of the colon is a very aggressive tumor that, despite clinical intervention, leads to a poor patient outcome. A differential diagnosis of sarcomatoid carcinoma should be raised for endoscopic biopsy specimens showing lesions with spindle cell morphology. The diagnosis can be verified by immunostaining for cytokeratin.
